# The histopathological diagnosis of atypical meningioma: glass slide versus whole slide imaging for grading assessment

**DOI:** 10.1007/s00428-020-02988-1

**Published:** 2020-12-10

**Authors:** Serena Ammendola, Elena Bariani, Albino Eccher, Arrigo Capitanio, Claudio Ghimenton, Liron Pantanowitz, Anil Parwani, Ilaria Girolami, Aldo Scarpa, Valeria Barresi

**Affiliations:** 1 Department of Diagnostics and Public Health, Section of Pathology, University of Verona, Verona, Italy; 2 Department of Pathology and Diagnostics, University and Hospital Trust of Verona, Verona, Italy; 3 Department of Clinical Pathology, and Department of Clinical and Experimental Medicine, Linköping University, Linköping, Sweden; 4 Department of Pathology & Clinical Labs, University of Michigan, Ann Arbor, MI USA; 5 Department of Pathology, Wexner Medical Center, Ohio State University, Columbus, OH USA; 6 Division of Pathology, Central Hospital Bolzano, Bolzano, Italy; 7 ARC-Net Research Centre, University and Hospital Trust of Verona, Verona, Italy; 8 Department of Diagnostics and Public Health, Polyclinic G.B. Rossi, P.le L.A. Scuro, 10, 37134 Verona, Italy

**Keywords:** Atypical meningioma, Whole slide imaging, Digital, Recurrence, Reproducibility

## Abstract

Limited studies on whole slide imaging (WSI) in surgical neuropathology reported a perceived limitation in the recognition of mitoses. This study analyzed and compared the inter- and intra-observer concordance for atypical meningioma, using glass slides and WSI. Two neuropathologists and two residents assessed the histopathological features of 35 meningiomas—originally diagnosed as atypical—in a representative glass slide and corresponding WSI. For each histological parameter and final diagnosis, we calculated the inter- and intra-observer concordance in the two viewing modes and the predictive accuracy on recurrence. The concordance rates for atypical meningioma on glass slides and on WSI were 54% and 60% among four observers and 63% and 74% between two neuropathologists. The inter-observer agreement was higher using WSI than with glass slides for all parameters, with the exception of high mitotic index. For all histological features, we found median intra-observer concordance of ≥ 79% and similar predictive accuracy for recurrence between the two viewing modes. The higher concordance for atypical meningioma using WSI than with glass slides and the similar predictive accuracy for recurrence in the two modalities suggest that atypical meningioma may be safely diagnosed using WSI.

## Introduction

Traditional diagnostic pathology has been progressively influenced by technological advancement. Although light microscopy still represents the gold standard for histopathological diagnosis, whole slide imaging (WSI) systems, used to capture, transmit, and store digital images, have attracted growing interest. Digital slides may have many advantages over glass slides such as easy archiving, research, teaching, and remote diagnosis or consultation [1–4].

In April 2017, the US Food and Drug Administration (FDA) first approved WSI for primary diagnosis in surgical pathology [5]. At the same time, validation studies were published regarding the deployment of WSI systems in several diagnostic settings, e.g., intraoperative services, cytology screening, and subspecialty consultation [6, 7]. Moreover, recent systematic reviews have highlighted the diagnostic reliability of digital modality [8–10]. However, even in countries where pathology laboratories are equipped with digital scanners, WSI is still underutilized for routine diagnostic clinical work due to factors such as high cost, lack of system interoperability, safety concerns, and regulatory restrictions [11, 12].

Neuropathology is one of the areas that has benefited most from digital pathology. WSI has enabled access via teleconsultation to expert neuropathologists, for intraoperative examinations and primary diagnostics, independent of the geographical location of the sample [12, 13]. Nonetheless, some neuropathologists still appear to be reluctant to work fully digitally [11], partly due to the perceived limitations in the recognition of mitoses and nuclear details in whole slide images [11, 12, 14, 15].

Few studies have been published on the reliability of WSI in surgical neuropathology for primary diagnosis [12, 14, 15]. These limited studies have shown that WSI is not inferior to light microscopy and that this technology can be used for primary diagnosis of central nervous system (CNS) tumors safely, if it is handled by trained neuropathologists who are aware of limitations and possible pitfalls [12, 14–17].

Meningiomas are the most frequent primary tumors of the central nervous system [18] and are currently classified into fifteen histotypes and three grades of malignancy [19]. Histological grading of these tumors relies on several criteria, including mitotic index [19]. In particular, atypical (grade II) meningiomas are diagnosed in the presence of (1) a mitotic index ranging between 4 and 19 mitoses per ten high-power fields (HPF) of 0.16 mm^2^; or (2) brain invasion; or (3) at least three minor atypical criteria among spontaneous necrosis, patternless architecture (sheeting), small cells with high nuclear/cytoplasmic ratio, macronucleoli, and hypercellularity [19].

A previous study reported an agreement of 87% between two neuropathologists assessing the histological grade of 172 meningiomas on glass slides; the lowest concordance was encountered for grade II meningiomas due to disagreement in mitotic counts [20].

Although previous studies on the analysis of concordance between glass slides and WSI in neuropathology did include some meningiomas, none of these investigations focused specifically on the reliability of grading meningiomas using digital pathology [12, 14, 15]. For this reason, the aim of this study was to analyze and compare the inter- and intra-observer concordance in the diagnosis of atypical meningioma using glass slides and WSI.

## Materials and methods

### Ethical issues

This study was approved by Comitato Etico per la Sperimentazione Clinica delle province di Verona e Rovigo (protocol n. 40400, 2019/07/19).

### Cases

Thirty-five atypical meningiomas diagnosed between 2001 and 2016 were randomly selected from the files of the Unit of Anatomic Pathology of the University and Hospital Trust of Verona, Italy.

The lead author (observer #1) served as the study coordinator and reviewed all hematoxylin and eosin–stained (H&E) slides to select a single representative diagnostic slide for each case [14]. The selected glass slides were de-identified.

### Histopathological assessment of glass slides

After a washout period of three weeks, observer #1 assessed major (mitotic index, brain invasion) and minor atypical criteria (sheeting, macronucleoli, spontaneous necrosis, hypercellularity, and small cells with high nuclear/cytoplasmic ratio), on each representative glass slide using a Nikon Eclipse 80i light microscope with a × 10/22 mm micrometer eyepiece. One additional senior pathologist (observer #2) and two residents in Anatomic Pathology (observers #3 and #4), all blinded to the original grading of these meningiomas, independently carried out the same assessment using the same light microscope.

Mitotic index was assessed counting mitoses in ten consecutive HPFs, in mitotic active areas. Then, the counts were normalized to obtain values in the equivalent of 1.6 mm^2^. According to the WHO (World Health Organization) criteria for meningioma grading [19], cases with ≥ 4 mitoses/1.6 mm^2^ were classified as having a high mitotic index. Brain invasion was defined by the presence of irregular tongue-like protrusions of tumor cells in the brain, without intervening leptomeninges [19]. Hypercellularity was defined by the presence of > 53 nuclei in the diameter of a HPF of 0.16 mm^2^ [21], which corresponds to > 76 nuclei using a light microscope with a × 10/22 mm micrometer eyepiece. Macronucleoli were defined as nucleoli visible under a × 10 objective lens and in ≥ 50% of the tumor [22, 23]. Sheeting was defined by the lack of whorls, lobules, syncytia, or small aggregations in ≥ 50% of the tumor [22, 23]. Spontaneous necrosis was defined by the presence of necrotic foci separated from surrounding viable tumor by a rim of pyknotic nuclei [22, 23].

### Histopathological assessment of WSI

**Fig. 1:**
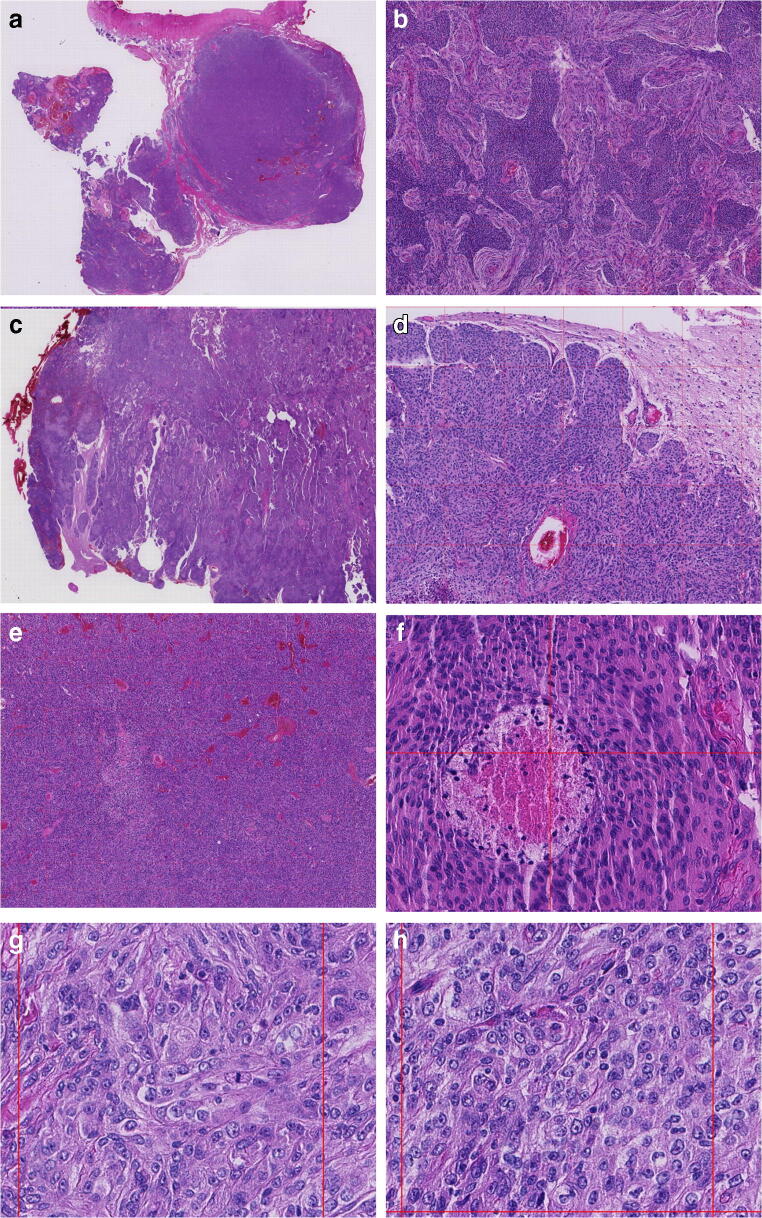
Atypical meningiomas captured on WSI. **a** Low-power view of an atypical meningioma, with its dural attachment. **b** Small cells with high nuclear/cytoplasmic ratio. **c**, **d** Brain invasion, with tongues of tumor cells infiltrating the brain parenchyma without intervening leptomeninges. **e** Sheeting with the absence of whorls or lobules. **f** Spontaneous necrosis showing gradual transition from the viable tumor, with a rim of pyknotic nuclei. **g** Mitosis in a squared cell corresponding to a field of 0.16 mm^2^. **h** Macronucleoli

The glass slides were scanned with a NanoZoomer S360 Digital slide scanner by Hamamatsu Photonics™. No data related to the patients were present on the slide label. The scanning was performed at × 40 magnification with seven z-stack levels and a 1.2-micron distance between each level. After scanning, the digital images were subjected to “deep focusing.” This procedure, generally used on cytological preparations [24], ensures a homogeneous vision of the tissue without blurring due to irregularity of the surface of the histological section or excessive thickness of the section itself. Finally, a zoomable grid made of squared cells was superimposed on the digital image, with each cell having an area of 0.16 mm^2^. After a washout period ranging from 3 to 6 weeks since the assessment of glass slides, all of the observers independently analyzed the histopathological features of meningiomas on WSI (Fig. 1). The mitotic index was assessed by counting mitoses in 10 consecutive 0.16 mm^2^ squared cells in mitotic active areas (Fig. 1). Hypercellularity was assessed by counting the nuclei in the row of a squared cell.

### Clinical data

Clinical records and registries were reviewed to retrieve information on the extent of surgical resection and development of recurrences. Recurrence was defined as the identification of a tumor at the site of previous complete surgery by means of computerized tomography or magnetic resonance imaging.

### Statistical analyses

Each case was classified as atypical for major criteria (high mitotic index and/or brain invasion), atypical for minor criteria (sheeting, macronucleoli, spontaneous necrosis, hypercellularity, and small cells with high nuclear/cytoplasmic ratio), or non-atypical, for each observer and in each viewing mode.

For each histological parameter and final diagnosis (atypical or non-atypical), the following measures were calculated: (1) inter-observer concordance within each viewing mode (glass slide and WSI); (2) intra-observer concordance between the different viewing modes; (3) predictive accuracy on recurrence (i.e., the accuracy to distinguish between the presence and absence of recurrence), using the area under the receiver operating characteristic curve (AUC). A probability (*P*) value less than 0.05 was considered significant. Statistical analysis was performed using the MedCalc 12.1.4.0 statistical software (MedCalc Software, Mariakerke, Belgium).

## Results

### Histopathological assessment using glass slides

**Fig. 2:**
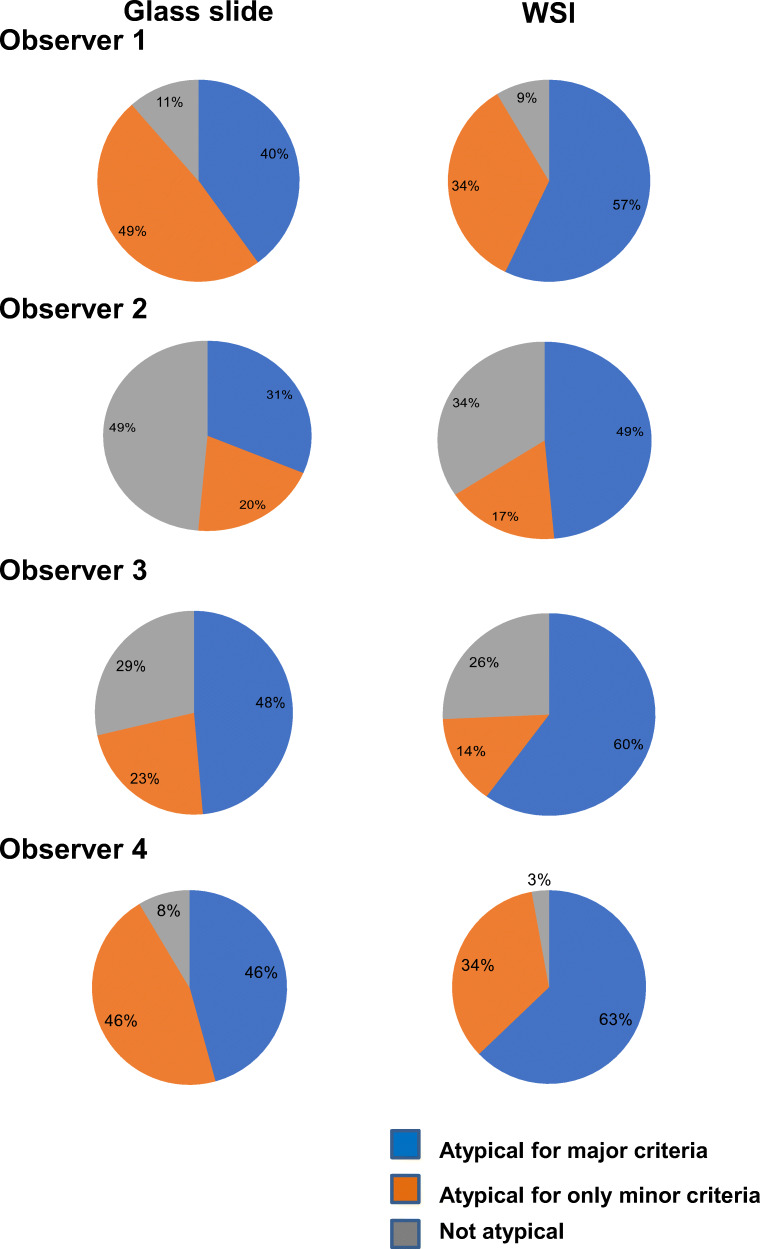
Classification of meningiomas as atypical for major criteria, atypical for only minor criteria, and not atypical, by four observers on glass slides and WSI**.** All observers classified a higher percentage of cases as atypical for major criteria, and a lower one as not atypical, on WSI compared to glass slide

Using the selected representative slides, observer #1 classified 31 (89%) meningiomas as atypical and 4 (11%) as non-atypical (grade I). Fourteen cases were atypical for major criteria (mitotic index ≥ 4/10HPF and/or brain invasion) and 17 were atypical for minor criteria (Fig. 2). The inter-observer concordance for atypical meningiomas was 54% (19/35 cases) (Table 1; Fig. 2). All observers agreed that 12 meningiomas were atypical for major criteria, 2 were atypical for minor criteria, and 2 were non-atypical (grade I). Three cases were atypical for major criteria for one or more observers and atypical for minor criteria for the others. The 16 discordant cases were rated atypical for minor (10 cases) or major criteria (6 cases: 2 showing brain invasion and 4 displaying high mitotic index for only some observers) by at least one observer, and not atypical (grade I) by the others.

**Table 1:** Inter-observer concordance for atypical meningioma and individual histopathological features, on glass slides and WSI

	Glass slide		WSI	
	All observers (%)	Senior pathologists (%)	All observers (%)	Senior pathologists (%)
Atypical meningioma	54	63	60	74
Atypical for major criteria	69	86	80	86
Atypical for minor criteria	46	60	63	77
Brain invasion	83	97	89	97
High mitotic index	80	86	69	80
Hypercellularity	74	77	86	86
Sheeting	57	74	66	77
Macronucleoli	37	49	40	51
Small cells	34	49	34	49
Spontaneous necrosis	26	51	31	54

**Fig. 3:**
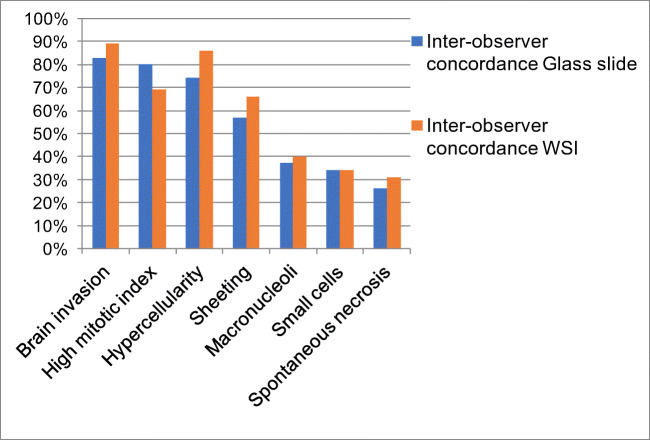
Inter-observer concordance for histopathological features required for meningioma grading on glass slide and WSI. Inter-observer concordance was higher on WSI than on glass slides, for all parameters with the exception of high mitotic index

**Fig. 4:**
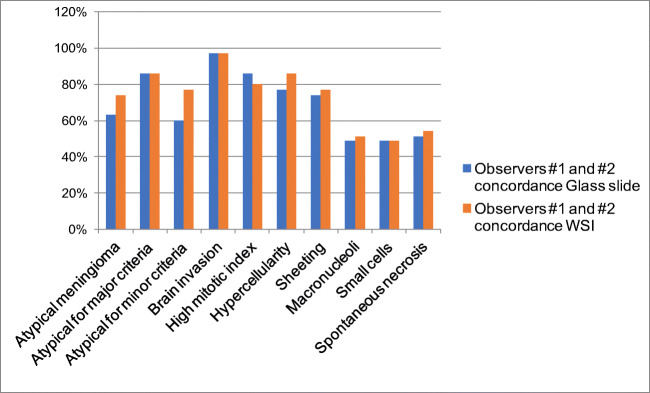
Concordance between senior pathologists for histopathological features required for meningioma grading on glass slide and WSI. Inter-observer concordance was higher on WSI than on glass slides, for all parameters with the exception of high mitotic index

With regard to single parameters, the highest concordance was achieved for brain invasion (83%; 29/35 cases), followed by high mitotic index (80%; 28/35), hypercellularity (74%; 26/35), and sheeting (57%; 20/35) (Table 1). Spontaneous necrosis had the lowest inter-observer concordance (26%; 9/35) (Fig. 3). The inter-observer concordance for atypical meningiomas between the two senior pathologists (observers #1 and #2) was 63% (22/35) (Table 1). The 13 discordant cases were classified as atypical for minor (8 cases) or major (5 cases) criteria by one observer, and not atypical (grade I) by the other. The highest concordance was achieved for brain invasion (97%; 34/35 cases), followed by high mitotic index (86%; 30/35), hypercellularity (77%; 27/35), and sheeting (74%; 26/35). Concordance ranged between 49 and 51% for the remaining parameters (Table 1; Fig. 4).

### Histopathological assessment using WSI

Using WSI, the inter-observer concordance for atypical meningiomas was 60% (21/35 cases) (Table 1) (Fig. 3). All observers classified 17 meningiomas as atypical for major criteria, 2 as atypical for minor criteria, while two cases were rated atypical for major criteria by one or more observers and atypical for minor criteria by the others (Table 2). The 14 discordant cases were rated atypical for minor (9 cases) or major (5 cases) criteria by at least one observer, and not atypical by the others.

**Table 2:** Number of meningiomas rated positive for each histopathological parameter by the four observers on glass slide and WSI

Parameter	Glass slide				WSI			
	Observer #1	Observer #2	Observer #3	Observer #4	Observer #1	Observer #2	Observer #3	Observer #4
Brain invasion	6	7	8	10	6	7	7	9
High mitotic index	10	5	10	9	17	12	20	15
Hypercellularity	1	7	3	1	1	4	0	0
Sheeting	29	24	25	19	30	24	27	25
Macronucleoli	24	6	23	11	26	11	27	11
Small cells	25	13	21	17	26	12	24	18
Spontaneous necrosis	20	15	28	16	21	15	28	18

**Table 3:** Intra-observer concordance for atypical meningioma and individual histopathological features between glass slides and WSI

	Observer #1 (%)	Observer #2 (%)	Observer #3 (%)	Observer #4 (%)	Median (%)
Atypical meningioma	91	86	74	94	89
Brain invasion	100	91	86	97	94
High mitotic index	80	79	77	71	78
Hypercellularity	94	82	97	91	93
Sheeting	97	97	77	94	96
Macronucleoli	94	82	100	83	89
Small cells	97	94	97	91	96
Spontaneous necrosis	97	91	94	94	94

The highest concordance was reached for brain invasion (89%; 31/35 cases), followed by hypercellularity (86%; 30/35), high mitotic index (69%; 24/35), and sheeting (60%; 21/35) (Table 1; Figs. 4 and 3). Spontaneous necrosis had the lowest inter-observer concordance (31%; 11/35).

Between the two senior pathologists (observers #1 and #2), the inter-observer concordance for atypical meningioma was raised to 74% (26/35). The 9 discordant cases were classified as atypical for minor (6 cases) or major (3 cases) criteria by one observer, and not atypical by the other (Table 1). The highest concordance was reached for brain invasion (97%; 34/35 cases), followed by hypercellularity (86%; 30/35), high mitotic index (80%; 28/35), and sheeting (77%; 27/35). Concordance ranged between 49 and 54% for the remaining parameters (Table 1; Fig. 4).

### Concordance between glass slide and WSI

**Fig. 5:**
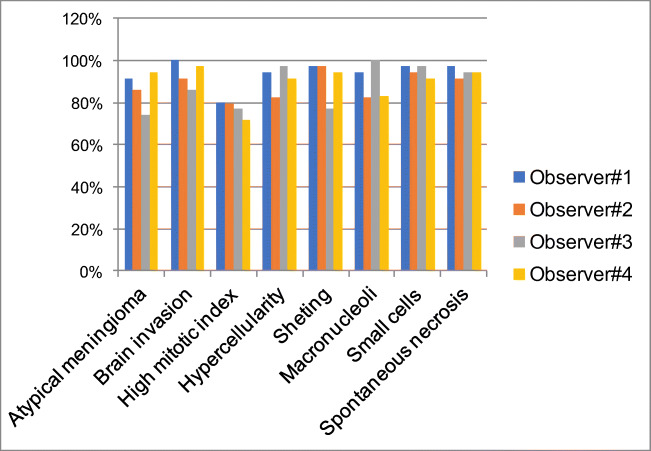
Intra-observer concordance for atypical meningioma and individual histopathological parameters between glass slide and WSI. For all four observers, high mitotic index had the lowest intra-observer concordance

The intra-observer concordance between glass slides and WSI reached at least 70% for all parameters and all observers (Tables 2 and 3; Fig. 5). The lowest intra-observer concordance was achieved for high mitotic index (range: 71–80%; median: 78%) and the highest for sheeting (range: 77–97%; median: 96%) and small cells (range: 91–97%; median: 96%). All four observers classified more cases as atypical for high mitotic index using WSI compared to glass slides (Fig. 2; Table 2). Total 11 cases were rated discordantly for high mitotic index by two senior pathologists. Nine had a mitotic index of 4–6/1.6 mm^2^ in WSI and of 5/10 HPFs (equal to 3.4/1.6 mm^2^) in glass slides. In 2/11 cases, mitotic index was overestimated in WSI.

### Predictive accuracy of glass slides and WSI histopathology for recurrence

All cases underwent complete surgical resection and 25/35 (71%) developed a recurrent tumor. High mitotic index was the parameter associated with the highest AUC value for prediction of recurrence for three of four observers using glass slides, and for all observers using WSI (Table 4). The predictive accuracy of high mitotic index, brain invasion, and sheeting increased using WSI rather than glass slides (Table 4); however, AUC was not significantly different between the two viewing modes for any parameter and any observer.

**Table 4:** Area under the receiver operating characteristic curve (AUC) of histopathological parameters for prediction of recurrence, on glass slides and WSI

Parameter	Observer #1		Observer #2		Observer #3		Observer #4	
	AUC glass slide	AUC WSI	AUC glass slide	AUC WSI	AUC glass slide	AUC WSI	AUC glass slide	AUC WSI
Brain invasion	0.50	0.50	0.51	0.51	0.53	0.55	0.50	0.55
High mitotic index	0.64	0.72	0.60	0.61	0.58	0.65	0.56	0.68
Hypercellularity	0.54	0.52	0.58	0.58	0.50	0.50	0.54	0.50
Sheeting	0.57	0.52	0.59	0.59	0.55	0.59	0.50	0.62
Macronucleoli	0.53	0.51	0.55	0.53	0.51	0.53	0.53	0.53
Small cells	0.55	0.51	0.63	0.61	0.54	0.53	0.52	0.54
Spontaneous necrosis	0.51	0.52	0.61	0.61	0.51	0.51	0.56	0.54

## Discussion

In this study, we assessed the inter- and intra-observer concordance in the diagnosis of atypical meningiomas using glass slides and WSI.

Our findings can be summarized as follows: (1) the inter-observer concordance for atypical meningioma was 54% on glass slides and 60% on WSI and, in both viewing modes it was related to the pathologists’ years of practice; (2) sub-optimal concordance rates were mainly related to low inter-observer agreement for minor atypical criteria; (3) the inter-observer agreement was higher when using WSI than with glass slides for all histopathological parameters, with the exception of high mitotic index; (4) this latter feature had the lowest intra-observer concordance between the two viewing modes, as all observers classified more cases as having a high mitotic index on WSI than on glass slides; and (5) the predictive accuracy of all histopathological parameters for recurrence was not significantly different between the two viewing modes.

Our findings confirm previous evidence [20, 25–28] that the assessment of histopathological features of atypical meningiomas is highly subjective, poorly reproducible, and linked to the observer's diagnostic experience, and demonstrate the same limitations using WSI.

In fact, the agreement for atypical meningioma among four observers, including two senior pathologists and two residents, was only 54% using glass slides and 60% using WSI, but increased to 63% and 74%, respectively, when only the two experienced pathologists were considered.

However, even these latter values are much lower than the 87% concordance rate previously reported between two neuropathologists grading 172 meningiomas employing conventional light microscopy [20]. This discrepancy may depend upon the inclusion of a high percentage (48.5%) of atypical meningiomas with only minor atypical criteria in this series. In fact, the concordance rates obtained for major atypical parameters in both viewing modes between the two senior pathologists were in line with those reported in the aforementioned study (97% and 97% vs 92.4% for brain invasion; 86% and 80% vs 79.1% for high mitotic index) [20], while those for macronucleoli (49% and 51% vs 76.7%), small cells (49% and 49% vs 79.1%), and spontaneous necrosis (51% and 54% vs 85.5%) were significantly lower [20]. It should be noted that the use of more standardized definitions for necrosis and macronucleoli did not lead to greater reproducibility in this study. However, minor atypical parameters were more reproducible with WSI and this resulted in greater agreement in the classification of meningiomas as atypical vs non-atypical, in this viewing mode compared to the use of glass slides.

In line with a previous study using light microscopy [20], brain invasion was the most reproducible parameter on both glass slides and WSI and was classified differently by the two senior pathologists in only one case. On the other hand, concordance for high mitotic index was sub-optimal in both viewing modes and worsened using WSI. In fact, among four observers, high mitotic index was discordant in 7 cases on glass slides and in 11 with WSI, and the same happened between the two experts, who were discordant in 5 cases using conventional microscopy and in 7 with digital slides.

However, the higher agreement between the two senior pathologists demonstrates that diagnostic experience is relevant in the recognition of mitotic figures in digital slides, as is the case with traditional light microscopy [26]. Disagreement in the assessment of mitoses may be related to several factors, including variability in the diligence and time spent in searching for mitotic figures [20] or discordance in the distinction between true mitoses and mimics such as apoptosis and karyorrhexis [29]. The advantage of using WSI to determine if a meningioma has a high mitotic index is that it is not necessary to normalize the mitotic count to a HPF of 0.16 mm^2^ as required by the WHO criteria [19]. Indeed, the grid of 0.16 mm^2^ squared cells superimposed on the digital image simplified the assessment in 10 HPF of this area. Nonetheless, as already reported [12], all raters in this study complained that the recognition of mitoses was more challenging on WSI than in glass slides. The difficulty in recognizing mitoses on WSI may be attributable to the lower contrast between chromatin and the nuclear background on digital slides, rendering the nuclei darker and hence more difficult to interpret, and/or to the inability to adjust the fine focus for potential mitotic figures [12]. Hopefully, the use of artificial intelligence systems could help overcome these limitations of digital pathology [30].

The median intra-observer concordance between glass slides and WSI was around or greater than 90% for all histopathological features, except for high mitotic index (78%), which was the least reproducible parameter using the two viewing modes. This is not surprising, as the evaluation of mitotic index was already reported as the main cause of diagnostic discrepancy between glass slides and WSI in other neuropathology studies [12, 14]. However, in such studies, there was a tendency to under-grade gliomas or meningiomas with WSI compared to glass slides, due to the under-recognition of mitoses using the first modality [12, 14]. In contrast, in this study, all observers classified more meningiomas as having a high mitotic index on WSI. In the majority of cases, this happened for meningiomas having a mitotic index close to the cut-off value of 4/1.6 mm^2^. However, in some cases, the mitotic count was overestimated in WSI as the observers considered chromatin condensation image as a mitotic figure on WSI, but not on glass slides. However, by doing so, the predictive value for recurrence was higher for high mitotic index assessed on WSI than on glass slides, albeit this difference was not statistically significant.

The good intra-observer agreement and similar predictive values of histopathological features in the two viewing modes demonstrate that meningiomas can be safely and accurately diagnosed using WSI. A possible limitation of this study could be that the coordinator, who selected the slides to be assessed, also served as an observer and could have been biased in the evaluation of grading. However, the leading pathologist was unaware of how the individual parameters had been assessed in the original diagnosis; in addition, demonstrating an unbiased judgment, she classified some of the cases as non-atypical.

In conclusion, this study shows that atypical meningioma may be safely diagnosed using WSI. The transition to this modality could simplify and standardize the assessment of mitotic index, without the need of normalization according to the microscope used. Although the inter-observer reproducibility of minor atypical criteria remains unsatisfactory, in this study, it was slightly higher using WSI compared to glass slides. Finally, the similar predictive value of all histopathological features when using the two different modalities further highlights the reliability of the diagnosis of atypical meningioma with WSI.

## Data Availability

Data will be available upon request to the corresponding author.
